# Influence of cephalexin on cadmium adsorption onto microplastic particles in water: Human health risk evaluation

**DOI:** 10.1016/j.heliyon.2024.e37775

**Published:** 2024-09-10

**Authors:** Madineh Khoshmanesh, Ali Mohammad Sanati, Bahman Ramavandi

**Affiliations:** a Department of Environmental Science, Persian Gulf Research Institute, Persian Gulf University, Bushehr, Iran; b Systems Environmental Health and Energy Research Center, The Persian Gulf Biomedical Sciences Research Institute, Bushehr University of Medical Sciences, Bushehr, 7518759577, Iran

**Keywords:** Microplastic, Polyethylene, Desorption, Cephalexin, Co-adsorption

## Abstract

This paper explores the impact of environmental factors on the adsorption of cadmium (Cd) and cephalexin (CEX) onto polyethylene (PE) microplastics. The study focused on Cd adsorption behavior on microplastics (MPs) of various sizes, revealing that particles sized 30–63 μm exhibited the highest adsorption capacity compared to other sizes. Cd sorption was significantly influenced by initial pH and salinity levels. Experimental data closely matched both the Langmuir (R^2^ > 0.91) and Freundlich (R^2^ > 0.92) isotherms. Cd adsorption onto PE particles was greater than CEX adsorption, with the maximum Cd uptake capacity measured at 1.8 mg/g. FTIR analysis indicated that Cd and CEX adsorption onto MPs was likely governed by physical interactions, as no new functional groups were detected post-uptake. The desorption rates of Cd and CEX from PE microplastics were evaluated in various liquids, including aqueous solution, tap water, seawater, and synthetic gastric juice. The health risks associated with Cd, in combination with MPs and CEX, for both children and adults were assessed in groundwater and aqueous solutions. This study offers scientific insights and guidelines for examining the environmental behavior, migration, and transformation of microplastics and their related ecological risks in scenarios of combined pollution.

## 1 Introduction

Plastics in the packaging industry help people's health and safety, as well as save energy, and have many general benefits [1,2]. Accumulation of waste by human societies into the ocean [3], plastics are among the wastes with about 92 %, leading to the pollution of beaches and waters [4]. In addition to reducing the landscape and beauty of the ecosystem, this pollution can also affect public health and biodiversity [5,6]. The durability of plastic waste makes it resistant to decomposition, but exposure to ultraviolet (UV) rays, mechanical wear, temperature changes, and biological decomposition can weaken its structure and cause it to break down into smaller particles [[7], [8], [9], [10]]. Since the beginning of the 20th century, microplastics (MPs) have been considered pollutants [11]. There is an accumulation potential for coastal and marine areas of 250 metric tons (Mt) by 2025 [7], especially polyethylene (PE) and polypropylene (PP) microplastics, which have the highest amount of production [12]. Freshwater ecosystems (rivers about 70–80 %) play a role in the transfer of microplastics [5,13]. Recently, the widespread presence of plastic materials in the environment has emerged as a significant issue [14]. Concerns such as the non-renewability of plastic, the accumulation of persistent pollutants on plastics, the durability of plastic against destruction– mortality, and physical damage to aquatic organisms due to eating plastics [8,15,16].

In a complex aquatic ecosystem with different pollutants [17], MPs, as carriers of toxic pollutants can increase the effect of chemical pollutants and also grow the bioavailability of these contaminants [18]. The small dimension of microplastics is certainly a critical factor to consider when discussing their negative effects [19], it can negatively impact fish swimming performance by reducing their ability to swim effectively and more pollutant absorption [20,21]. Plankton inadvertently consume microplastics (MPs), which then become integrated into their tissue [22]. The hydrophobic surface of microplastics also attracts contaminants [18,23]. PE and polystyrene (PS) microplastics have the highest absorption capacity compared to other ones in laboratory conditions [20]. Much research has been done on the absorption of heavy metals, persistent pollutants, pesticides, polycyclic aromatic hydrocarbons, cosmetic health products, and drugs in MPs [18,23]. Microplastics enter the bodies of marine creatures with absorbed contaminants and can cause additional toxicity [24]. The existence of drugs in waters [22], the lack of legal standards for the disposal of drugs, the increase in their consumption in recent years [25], and the simultaneous presence of heavy metals and antibiotics in natural waters [26] have led to microplastic contamination with these pollutants in the aquatic medium [22,27]. Complex pollutants (metals and antibiotics) are more toxic and can hurt bacteria and fungi, which are an integral part of the ecosystem [28].

Earlier research using Energy Dispersive X-ray Spectroscopy (EDX) identified the presence of heavy metals, including cadmium (Cd), lead (Pb), copper (Cu), zinc (Zn), nickel (Ni), and iron (Fe), associated with microplastics [8,29]. The equilibrium partition coefficient (K_d_) for metals ranked from 4 to 220 L/kg [8]. In research, an energy dispersive X-ray analysis (EDX) analysis showed the adhesion of Fe and Cr metals to the PE surface [30]. Purwiyanto et al., have found that the level of lead and copper in microplastics was higher compared to the water [31]. Chen et al. [32] investigated the uptake of tetracycline antibiotics including tetracycline hydrochloride, chlorotetracycline hydrochloride, and oxytetracycline in a PE in water and concluded that the adsorption capacity of tetracycline was 53.52 ± 3.43 μg/g. Antibiotic adsorption almost happens at the PE surface through non-bonded interactions and van der Waals force [32].

In this study, the adsorption and desorption of cephalexin (CEX) antibiotic and Cd on the surface of polyethylene microplastics are discussed. Cd is a heavy metal that is commonly found in the environment [33,34]. The cephalexin antibiotic was chosen due to its worldwide usage and presence in aquatic ecosystems [35]. CEX is one of the beta-lactam antibiotics with a structure similar to penicillin [36,37]. The primary objective of this study was to investigate the adsorption and desorption of cadmium (Cd) and the antibiotic cephalexin (CEX) onto polyethylene (PE) in various aquatic environments, both individually and in combination. The effects of environmental factors such as salinity, contact time, pH, and plastic particle size were analyzed, along with the adsorption kinetics and isotherms. Additionally, the impact of cadmium in conjunction with microplastics and CEX on health risks was assessed for groundwater and aqueous solution samples.

## 2 Materials and methods

### 2.1 Materials

Heavy polyethylene microplastic powder was prepared from Mehr Petrochemical Company (Asalouye, Iran), and its different sizes were obtained with a sieve shaker. Table 1 shows the weight percentage of microplastic in each microplastic size. Other chemicals, including the antibiotic cephalexin, were supplied by Farabi Pharmaceutical Company (Isfahan, Iran), while Cd(NO_3_)_2_·4H_2_O was obtained from Merck. Pepsin A (35 kDa) was sourced from Sigma-Aldrich.

**Table 1:** The weight percentage of microplastics for each size within a 361.18 g sample.

Size (μm)	Microplastic (w.%)
>500	0.76
250–500	3.40
125–250	51.30
63–125	35.20
30–63	8.10
<30	1.02

### 2.2 Preparation of cadmium and cephalexin solution

To prepare the Cd stock solution (100 mg/L), 2.74 g of Cd(NO_3_)_2_·4H_2_O was dissolved in 1 L of distilled water and the rest of the concentrations (3, 5, 10, 15, and 20 mg/L) were obtained by dilution method. The exact amount of 0.01 g of cephalexin was accurately weighed with a scale and dissolved in 10 mL of water, and other concentrations were achieved by diluting the main solution. In addition, the CEX solution was kept at a temperature of 2 °C. As this solution is unstable, therefore, for each test series, the desired concentration was prepared immediately.

### 2.3 Characterization

The surface morphology was investigated using an Atomic Force Microscopy analysis (AFM, Brisk, ARA Research, Iran) to track small changes in the microplastic surface before and after Cd and CEX adsorption. Individual microplastic particles were fixed to a glass slide using double-sided adhesive. Fourier transform infrared spectroscopy (FTIR4600, Jasco, Japan) was employed to characterize the functional groups of polyethylene microplastics both before and after adsorption with Cd and CEX. The Brunauer, Emmett, and Teller (BET, ASAP2020, Micromeritics, USA) method was utilized to determine the pore area and volume of the polyethylene microplastics through nitrogen adsorption-desorption at 77 K. Additionally, for thermal gravimetric analysis (TGA) test, 10 mg of PE were scanned from 30 to 600 °C at a heating rate of 10 °C per minute using an instrument (STA6000, PerkinElmer, USA). Also, PE samples were analyzed using powder X-ray diffraction (XRD, D8 Advance BRUKER Germany). To determine the zero point charge pH (pHzpc), 100 mL of distilled water containing 0.1 M sodium chloride was added to seven different containers, with the initial pH adjusted to range from 3 to 9. Next, 0.5 g of polyethylene microplastic particles sized 30–63 μm were introduced into each container and shaken for 12 h at 150 rpm. Afterward, the final pH was measured, and a graph plotting "initial pH - final pH” against the initial pH was created. The point where the line intersected the X-axis was identified as the pHzpc [38,39].

### 2.4 Procedure of experiments

The initial tests were carried out to determine the effect of dose, size, salinity, pH, the initial content of metal ions and antibiotic, and the contact time on Cd adsorption onto microplastics. The PE sizes (30–250 μm) were selected after sizing, which are the usual sizes identified in aquatic environments [24] and had the greatest adsorption of pollutants. Precisely 0.1 mg of microplastics and 100 mL of 1 mg/L Cd solution were placed into an Erlenmeyer flask and transferred to a shaker set at 200 rpm with a stable room temperature of 25 ± 1 °C. The variables of the investigation were pH (4–8), salinity (0–40 g/L), microplastic quantity (0.4–1.4 g/L), the initial content of cadmium (1–10 mg/L), cephalexin (3–10 mg/L), and the contact time (5–300 min). The conditions of all runs are listed in Table 2. All tests were replicated and done at the temperature of 25 ± 1 °C. After filtering with a 0.22 μm filter, the concentration of the remaining metal and antibiotic was measured. The cadmium ions residual was determined with an atomic absorption device (PG/AA500-England), and the antibiotic concentration was measured at the wavelength of 261 nm with a spectrophotometer (Shimadzu, Model UV 1700) [40]. In the experiments, 0.1 M NaOH (3.99 g/L) and 0.1 M HCl (3.64 g/L) solutions were applied to regulate the water pH.

**Table 2:** Test conditions and variables.

Run of test	Variable						
	Size (μm)	pH	Salinity (g/L)	PE dose (g/L)	CEX (mg/L)	Cd (mg/L)	Time (min)
Effect of particle size on the Cd sorption onto PE	30-63, 63–125, 125–250, 250-500	7	0	1	0	1	90
Effect of water pH on the Cd sorption onto PE in single and binary systems (Cd, CEX)	30–63	4, 6, 7, 8	0	1	3	1	90
Effect of water pH on the CEX uptake by PE	30–63	4, 6, 7, 8	0	1	3	0	90
Effect of salinity on the Cd sorption by PE	30–63	7	0, 20, 40	1	0	1	90
Effect of PE dose on the Cd sorption by PE	30–63	7	0	0.4, 0.6, 0.8, 1, 1.2, 1.4	0	1	90
Effect of CEX concentrations on the Cd sorption by PE	30–63	7	0	1	0, 3, 5, 10, 15, 20	1	90
Isotherm study	30–63	7	0	1	0	1	90
					3		
Kinetic study	30–63	7	0	1	0	1	5, 10, 20, 45, 90, 180, 300
					3		

### 2.5 Data analysis

The following relationship (Eq. (1)) was used to measure adsorption percentages:

(1) R%=[(C˳−C)/C˳]×100

C_0_ (mg/L) is the initial level of the metal or antibiotic and C (mg/L) denotes pollutant concentration after adsorption into microplastics. The illustrations were depicted using Origin 2022 software.

### 2.6 Adsorption kinetic and isotherm equations

Two kinetic models, the pseudo-first-order equation (PFO, Eq. (2)) and the pseudo-second-order (PSO, Eq. (3)), were utilized to analyze and fit the obtained data.

(2) $$qₜ=qₑ\left[1-\exp\left(-k_{1}t\right)\right]$$

(3) $$qₜ=\frac{k₂qₑ\;²t}{1+qₑk₂t}$$

Where q_e_ (mg/g) and q_t_ (mg/g) denote the level of adsorbed pollutant at equilibrium and time t (min), and k_1_ (L/min), k_2_ (g/mg^**.**^min) reflect the rate constants of the pseudo-first-order and pseudo-second-order, respectively.

To better describe the adsorption data, two widely used isotherms, Langmuir (Eq. (4)) and Freundlich (Eq. (5)), were employed [19].

(4) $$qₑ=K_{F}C{ₑ}^{1/n}$$

(5) $$qₑ=\frac{qₘK_{L}Cₑ}{1+K_{L}Cₑ}$$

Where q_e_ is the adsorbed metal concentration (mg/g), K_F_ is the Freundlich adsorption constant, *K*_*L*_ is the Langmuir constant linked to sorption capacity (mg/g), *C*_*e*_ is the content of adsorbate at equilibrium point (mg/L), n is the non-linearity coefficient, and q_m_ is maximum sorption capacity (mg/g) [41].

The test conditions for kinetic and isotherm study are presented in Table 2.

### 2.7 Desorption test

Desorption tests were performed on a certain amount of PE microplastics (0.1 g/L), cephalexin (3 mg/L), and cadmium (1 mg/L). To conduct the experiments, first, the adsorption tests were performed in distilled water, then the amount of desorption was evaluated in environments such as distilled water (pH: 6.91), seawater (pH: 8.34), city tap water (pH: 7.35), and simulated gastric juice. To simulate the gastric juice of warm-blooded animals, the amount of 3.2 mg/L of Pepsin A was poured into a solution of 100 mM of sodium chloride salt, and its pH was adjusted to 4 [42]. The temperature of the tests for all environments was 25 °C, except for the gastric juice, which was done at 37 °C. In this section, cadmium and CEX desorption from the microplastics was calculated.

## 3 Results and discussion

### 3.1 Characterization

**Fig. 1:**
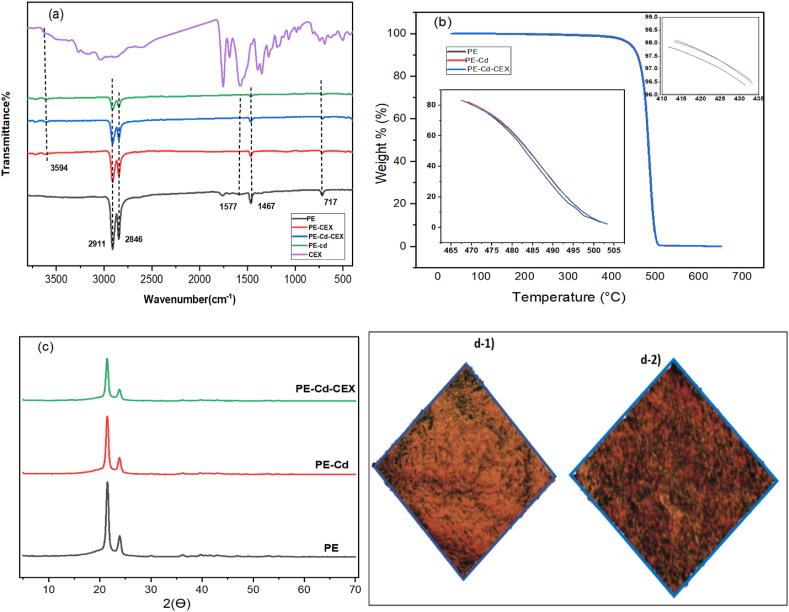
(a) FTIR, **(b)** TGA, and **(c)** XRD images of polyethylene microplastic before and after adsorption **(d)** AFM images of microplastic **(d-1)** before and **(d-2)** after adsorption. In these images, the size of microplastic particles was 30–63 μm.

In this study, the initial test focused on assessing the impact of different microplastic sizes on pollutant adsorption. The 30–63 μm size range demonstrated the highest adsorption, leading to its selection for exploring the effects of other parameters. Furthermore, particles within this size range were examined for their properties, as they had the most significant influence. The FTIR of PE-microplastics before and after exposure to cadmium ions and cephalexin is depicted in Fig. 1a. The FTIR image of fresh polyethylene shows functional groups of C-H vibration (2700-3000 cm^−1^), C=C vibration (1550-1718 cm^−1^), in-plane C-H vibration (1400-1500 cm^−1^), and out-of-plane C-H vibration (650-730 cm^−1^) [33,41,43,44]. The FTIR test revealed that there were no new peaks observed following the adsorption of the cephalexin antibiotic, which could be due to the lack of covalent bond formation after the process. The presence of intramolecular van der Waals forces during the adsorption of CEX and Cd ions onto PE was further confirmed, similar to the results of Chen's research on tetracycline sorption by PE [32]. In this research, like FTIR analysis in the other studies, no new peak was formed after metal uptake by PE, thus, physical interactions are responsible for adsorption [41,45].

Fig. 1b illustrates the TGA results obtained for the fresh and reacted PE microplastics. After the adsorption of cadmium and cephalexin, the initial weight loss of polyethylene increased from 421.55 °C to 425.05 °C and 425.3 °C, respectively. This suggests that the polyethylene samples containing pollutants exhibited slightly greater thermal stability compared to the fresh ones [46].

The XRD test results, shown in Fig. 1c, display two peaks for polyethylene microplastics at 2Ɵ values of 21.5° and 23.9°. Following pollutant adsorption by the microplastics, the peak positions shifted slightly to 21.4° and 23.8°, respectively. Additionally, the intensity of the peaks decreased slightly after the adsorption of cadmium and cephalexin. These findings suggest that the crystallinity of the PE microplastics decreased after adsorption. You et al., 2021 stated that the XRD of the aged polyethylene particles has been affected by radiation, with a slight change in angle and a decrease in intensity [47]. In the research conducted by Zhang et al., 2020, the presence of Cr(VI) in low concentrations did not cause any wide change in the crystal structure of PE microplastics [48].

The AFM technique was used to characterize the surface properties of polyethylene microplastics before and after Cd and CEX adsorption (Fig. 1d). The surface of both images is almost the same, but the microplastic surface looks slightly darker after adsorption.

The N_2_ adsorption-desorption technique was applied to analyze the BET areas of the polyethylene samples before and after adsorption (Table 3). The results showed a low number of pores in both polyethylene microplastics. The BET area of the polyethylene microplastics decreased from 1.0635 m^2^/g to a level that was lower than the detection limit of the device [49]. The lower specific surface in this case is due to the accumulation of pollutants on the microplastic surface, which prevents nitrogen molecules from reaching the entire surface.

**Table 3:** Physicochemical properties of PE microplastics used in this study.

Size	Surface area	Micropore volume	Pore size	Density
**30**–**63 μm**	1.0635 m^2^/g	0.000663 cm³/g	5.9 nm	0.952 g/cm^3^

### 3.2 Effect of experimental conditions

#### 3.2.1 Microplastic size

**Fig. 2:**
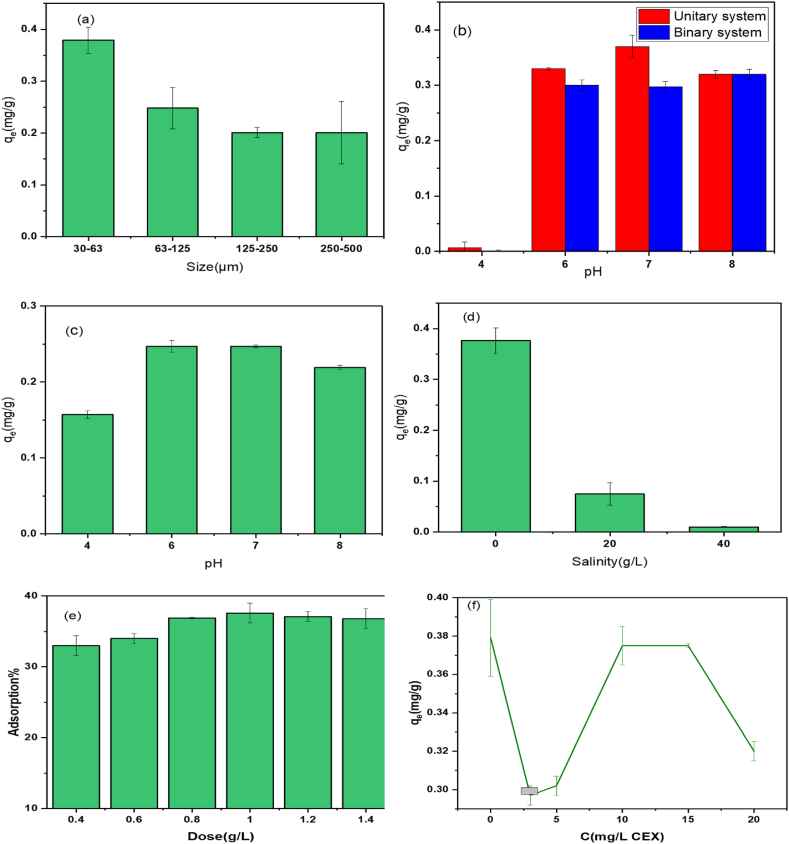
Influence of **(a)** particle size on the sorption of Cd by PE, **(b)** water pH on the uptake of Cd onto PE in a single system and binary system (Cd, CEX), **(c)** solution pH on the uptake of CEX by PE **(d)** salinity on the uptake of Cd by PE, **(e)** MP dose on the sorption of Cd by PE, and **(f)** different concentrations of cephalexin on the sorption of Cd by PE (pH 7, salinity: 0, PE size: 30–63 μm, dose: 0.1 g/L PE, Cd: 1 mg/L).

From the total weight of floating plastics in the oceans, about 13 %–32 % of particles include microplastics with a size of 30–500 μm [50]. Based on Fig. 2a, particles with smaller sizes have more surface area and there are more adsorption sites, so it can be the reason for the higher adsorption percentage of 30–63 μm size compared to other ones. These findings are consistent with previous studies that adsorption sites for the metal in microplastic with smaller sizes were more [47,51]. Fig. 2a shows the adsorption capacity of cadmium metal by microplastic with the lowest size is 0.379 mg/g. Contaminants are more probably to be transmitted by microplastics of a lesser size [52]. Conversely, in an investigation, the adsorption capacity reported a positive correlation with the size [20]. In this case, the larger size of the microplastic reaches equilibrium in a longer time [20]. Particles with a size of fewer than 10 μm had a more toxic effect on aquatic species than larger ones [53]. Adsorption capacity can be affected by environmental factors. Specific characteristics of microplastics such as pore volume, contact surface, pseudo-rubber characteristics of polyethylene, and several other factors can affect the adsorption capacity of microplastics [38].

#### 3.2.2 pH

The water pH is another parameter with a vital influence on the sorption of metal ions onto MPs [54,55]. The pH effect is linked to the charge of the sorbent and the type of metal ions [54]. Fig. 2b shows that the highest percentage of cadmium adsorbed at pH 8 similar to the results obtained by other researchers that the maximum sorption capacity at pH 7.06 was equal to 30.5 μg/g [33,56]. Holmes et al. reported that with the increase in the pH of the river water, there is an increase in the adsorption of cadmium, cobalt, nickel, and lead into microplastics [57]. These findings agree with ours, which reported the adsorption of chromium decreased and the adsorption of copper was almost constant [57]. These results demonstrate that pH can alter the surface of the morphology of microplastic and the charge of metal ions. A decrease in the competition for H^+^ and an increase in the electrostatic force with increasing water pH can cause an increase in the sorption of the cationic agent [17]. The study also determined that the pHzpc of polyethylene microplastic particles is 4.6. These microplastic particles carry a negative charge at pH levels above this value, which facilitates the adsorption of positively charged cadmium ions. Consequently, the efficiency of cadmium adsorption is higher at pH values greater than 4.6. Indeed, at high pH levels, there is a formation of Cd(OH)^+^ or Cd(OH)_2_ precipitates, which leads to a reduction in the adsorption value [58].

Antibiotics are ionizable compounds and have different ionization constants due to different functional groups [59]. Cephalexin exists in a cationic form at pH values of 2.56 and 6.88, in a zwitterionic form within the pH range of 2.56–6.88, and in an anionic form at pH levels above 6.88 [28,60]. As can be seen in (Fig. 2c) the maximum adsorption is at pH 6 and 7, and sorption decreases at pH 8 due to the anionic form of cephalexin and electrostatic interaction. Sun et al.'s research showed a decrease in the adsorption of norfloxacin in polyethylene at a pH > 9 [61]. In another investigation, there is an increase in the adsorption of sulfamethoxazole in polyethylene at pH 7.6 and a decrease in adsorption above 7.6 [62].

Fig. 2b illustrates the impact of pH on a system in which cadmium metal and cephalexin antibiotics are in contact with microplastics. At first, with the increase in pH level from 4 to 7, the sorption capacity increased and then decreased. In solutions with low pH (less than 3), H^+^ and H_3_O^+^ compete for adsorption sites [63]. At a pH lower than 6.88, a cationic form of cephalexin exists in the solution, which can slightly reduce cadmium adsorption by competing with cadmium for adsorption sites. But in the presence of cadmium in the form of Cd(OH)^+^, Cd(OH)_2_, and the anionic form of cephalexin at a pH above 6.88, competitive adsorption becomes weak. Meanwhile, the surface of polyethylene microplastic is negative at the pH of the common water environments [64], which helped to attract Cd^2+^ with positive charges. However, when the pH is acidic, the surface becomes positive and repels Cd^2+^. Also, the adsorption of Cd decreased because of the higher concentration of H_3_O^+^ in the solution that competed with Cd^2+^ for the adsorption sites. Thus, the combined effect of these pollutants and their transfer at different pHs should be considered [61].

#### 3.2.3 Salinity

The influences of salinity on the uptake of Cd by PE are illustrated in Fig. 2d. With the elevation in the salinity value, the capacity of Cd adsorption by microplastics decreased. This trend is similar to those reported in the literature [31,65]. However, in the research by Holmes et al., chromium uptake in microplastics increased and copper and lead showed minimal adsorption in freshwater [57]. In our study, cephalexin did not adsorb onto microplastics in a seawater environment. When the chlorine complex is formed with Cd in the form of CdCl^+^, CdOHCl, CdCl_2_, CdCl_3_^−^, and Na and Cd compete for the adsorptive sites, the adsorption capacity decreases [17]. Furthermore, there is an increase in microplastic accumulation with an increase in salinity, leading to a decrease in the number of active adsorption sites [55,66].

#### 3.2.4 Microplastic dose

To determine the adsorption capacity, the quantity of adsorbent is a critical parameter [24]. As seen in Fig. 2e by growing the quantity from 0.4 to 1.4 g, the adsorption percentage reaches from 33 % to 37.9 %. This trend is similar to the findings of previous investigations on the adsorption of the antibiotic lofloxacin by microplastics [24] that with the rise in the amount of adsorbent, part of the adsorption sites remain empty and are not saturated [67]. The PE dose of 1 g/L was chosen as the dose with the most adsorption, which seems sufficient for adsorption.

#### 3.2.5 Adsorption of Cd with CEX by polyethylene microplastics

Adsorption of Cd on PE at a different level of CEX (0–20 mg/L) was studied to find out the effect of CEX. Based on Fig. 2f, the sorption capacity with the increase of cephalexin had different trends of decrease, increase, and constant at the CEX level of 0–3 mg/L, 3–10 mg/L, and 10–15 mg/L, respectively. Subsequently, there is a reduction again in the sorption level at the antibiotic content of 15–20 mg/L. However, the sorption power of polyethylene is influenced by the presence of cephalexin. Generally, it was found that there is a negative impact in the sorption of Cd ions onto PE in the presence of CEX and 3 mg/L CEX had the greatest adverse effect. Since other conditions are constant, when the concentration of cephalexin is the lowest, according to the ‘qe=(C0−Ce)×volummass’ formula, the amount of sorption sites are also low, and as a result, a smaller amount of cadmium is absorbed by microplastics. Therefore, to assess the effect of CEX on microplastics in sorption isotherms and kinetics, the CEX concentration of 3 mg/L was selected. This value is the closest among those studied to what is observed in natural water sources [36,68]. As the concentration of cephalexin increased from 5 to 10 mg/L, the amount of cadmium adsorbed onto microplastic particles also increased. This suggests that at this concentration, cadmium ions may compete with cephalexin molecules for adsorption sites on the microplastics. However, at concentrations between 10 and 15 mg/L, cadmium adsorption remained relatively stable and then began to decline. This decline is likely due to the high concentration of cephalexin molecules (20 mg/L) compared to cadmium ions (1 mg/L), which means most of the adsorption sites are occupied by the antibiotics. Thus, varying amounts of cephalexin influence cadmium adsorption onto polyethylene microplastic particles. This result is consistent with studies on the uptake of Cu(II) by aged polystyrene (PS) and polyvinyl chloride (PVC) [27], as well as the adsorption of tetracycline and ciprofloxacin onto two types of microplastics (PS and PVC) in the presence of cadmium and copper ions [69].

### 3.3 Adsorption isotherms

**Fig. 3:**
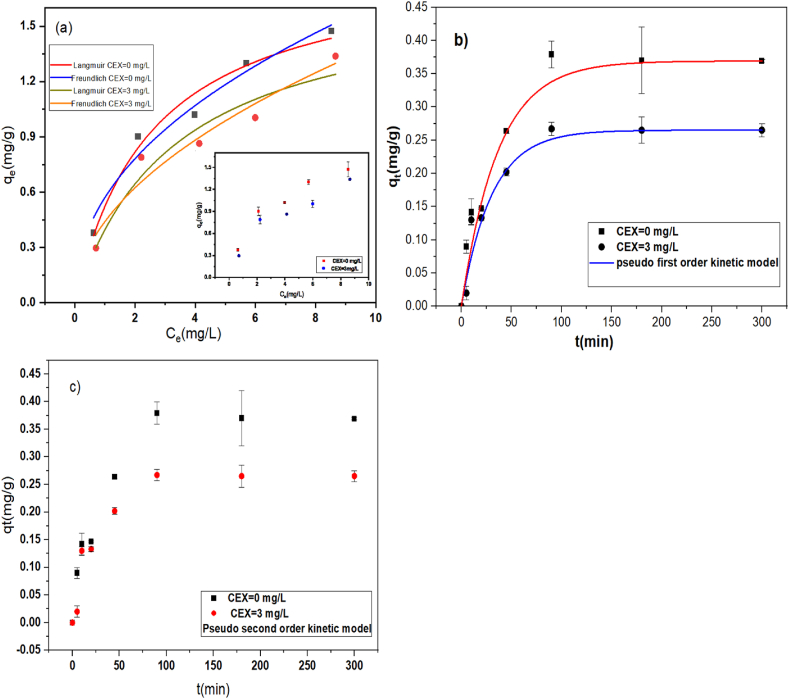
(a) Isotherms of Cd sorption on PE-microplastics with or without CEX and **(b**–**c)** kinetic data fitted by pseudo-first-order and pseudo-second-order (pH: 7, salinity: 0, PE size: 30–63 μm, dose: 1 g/L PE, Cd: 1 mg/L, CEX: 3 mg/L).

The equilibrium parameters have been computed for Langmuir and Freundlich isotherm models (Table 4) and their diagrams are provided in Fig. 3a. The experimental data for the adsorption of Cd(II) onto PE microplastics in the absence of CEX fit both models well, as indicated by the similar R^2^ values for the two models. This is consistent with findings reported in the literature [33], although the qm value is higher in this study. In another study, a significant amount of Pb (2316 μg/g) was adsorbed onto PE with high crystallinity [55,56]. When cadmium was adsorbed onto polyethylene in the presence of CEX, the R^2^ value for the Freundlich model was slightly higher than for the Langmuir model, and the n values were greater than 1, suggesting a physisorption process.

**Table 4:** Parameters of non-linear isotherms models.

Mixture	Langmuir				Freundlich		
	q_m_ (mg/g)	K_L_ (L/mg)	R_L_	R^2^	1/n	K_F_	R^2^
PE-Cd	1.86	0.38	0.27–0.72	0.964	0.45	0.57	0.953
PE-Cd-CEX	1.70	0.30	0.25–0.70	0.912	0.44	0.50	0.923

The q_m_ values for Cd adsorption onto polyethylene, both with and without CEX, are provided in Table 4. It was observed that the presence of 3 mg/L CEX limits Cd sorption by microplastics. Consequently, the qm value for adsorption with 3 mg/L CEX was lower compared to the value obtained without CEX.

### 3.4 Adsorption kinetics

The adsorption of metals by MPs can be explained by kinetic models [19]. Kinetic models of the reaction were investigated at a contact time of 300 min. Kinetics parameters for both pseudo-first and second-order models are listed in Table 5. The adsorption kinetic of Cd with and without CEX is shown in Fig. 3b and c. Based on the results, it is clear that there is a difference in the cadmium adsorption kinetics with and without 3 mg/L CEX. Assuming all conditions are constant, there is a negative effect of 3 mg/L CEX on Cd sorption by polyethylene. The calculated values for adsorption on PE showed that there is a relatively small difference between the R^2^ numbers of the models. Thus, the data of the investigation were described with both PFO and PSO models.

**Table 5:** Kinetic data fitted by non-linear models of pseudo-first-order and pseudo-second-order.

Mixture	Pseudo first order			Pseudo second order		
	q_e_ (mg/g)	k_1_	R^2^	q_e_ (mg/g)	k_2_	R^2^
PE-Cd	0.37	0.032	0.960	0.42	0.097	0.958
PE-Cd-CEX	0.26	0.038	0.954	0.297	0.162	0.950

In the previous study, the reaction kinetics for cadmium metal adsorption in microfibers that were naturally weathered followed pseudo-second-order [70]. In contrast, the sorption kinetics for heavy metals in degraded and pristine microplastics in seawater and river water is more consistent with the pseudo-first-order model [33,57]. The difference in surface adsorption kinetics is due to the difference in microplastics and reaction conditions [33].

### 3.5 Adsorption mechanism of cadmium and cephalexin to MP particles

Previous studies have explored the uptake of heavy metals into microplastics. The studies have reported electrostatic attraction, complexation, cation-π bond interaction, and physical sorption as the mechanisms for metal uptake on the microplastics in the water bodies. The main interaction (electrostatic attraction) is the interplay between cationic contaminants and microplastics owing to the negative charges at the MP surface in water resulting from the weak acid [56].

To further examine, FTIR analyses for the mechanism of CEX sorption by MPs were performed as illustrated in Fig. 1a. Functional groups of PE-MPs before and after cadmium uptake with or without CEX were identified to further study the mechanism. Based on the FTIR spectra, the wavenumbers of the characteristic peaks of microplastic before and after the uptake of pollutants are very similar. Due to the low uptake of Cd on the surface of polyethylene microplastics, very few alterations were recorded between the FTIR spectrum of polyethylene microplastics before and after Cd uptake. Hydroxyl functional groups at 3594 cm^−1^ after adsorption show that PE adsorbs CEX through hydrogen bonding. However, after adsorption, the peak of the carbon-carbon (C=C) disappeared.

**Fig. 4:**
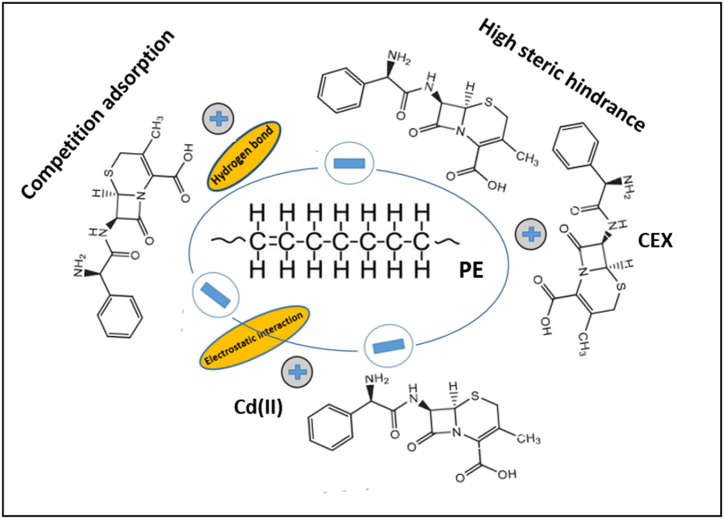
Simplified mechanisms for the impact of CEX on uptake of Cd by microplastics.

In this research, CEX is a zwitterionic and anion form and it shows that competition is created between CEX with Cd(II) for sorption on the negative sites on the PE microplastics [27]. In addition, there are complexes in neutral and acidic conditions owing to the affinity of Cd(II) toward CEX. As a result, the adsorption of Cd(II) would weaken because of the higher steric hindrance impact of the complexes of Cd-CEX. However, the three-component complex of Cd–CEX-MP has negative effects on the competition of CEX^+^ and Cd to be placed on the PE-microplastics (see Fig. 4). A similar report was also presented by Zhou et al., 2022 [27]. The adsorption of CEX and Cd onto polyethylene microplastics was found to be affected by the pH and ionic strength of the solution. The pHpzc of polyethylene is approximately 4.6. At pH levels above this value, the adsorbent's surface carries a negative charge, whereas below this pH, it has a positive charge. Consequently, at pH levels below the pHpzc, antibiotic anions are attracted to the positively charged surface due to electrostatic forces. In contrast, at pH levels above the pHpzc, the negatively charged surface repels the cephalexin anion, reducing its adsorption. As pH increases, the negatively charged surface of the microplastics favors the adsorption of Cd^2+^. Additionally, higher salinity decreases adsorption capacity due to cation exchange.

### 3.6 Desorption study

**Fig. 5:**
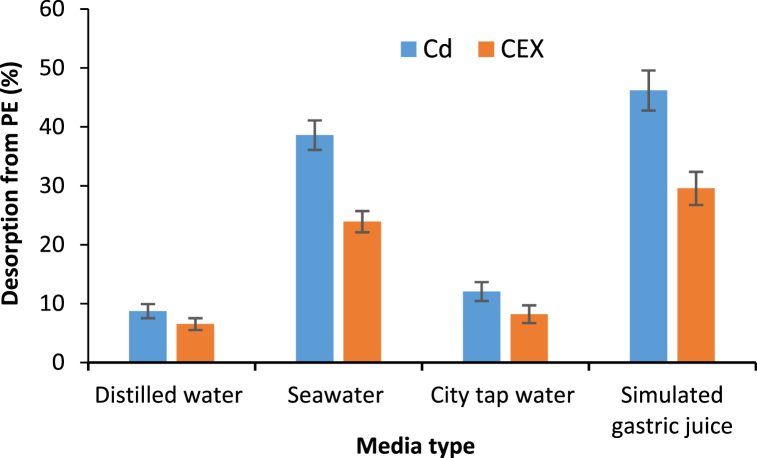
Desorption of cadmium and cephalexin from polyethylene microplastics (desorption time: 8 h, PE: 0.1 g/L, CEX: 3 mg/L, Cd: 1 mg/L, **distilled water**: pH: 6.91, temperature: 25 °C, **seawater**: pH: 8.34, temperature: 25 °C, **city tap water**: pH: 7.35, temperature: 25 °C, and **simulated gastric juice temperature**: pH: 4, 37 °C).

Microplastics may carry dangerous pollutants and increase toxicity in the environment. Accordingly, studying the release of pollutants from this pollutant carrier (which is itself a pollutant) can be useful [42,47]. Microplastics can enter the human body (via food) or enter water sources and harm microorganisms and aquatic organisms. Therefore, the release amount of pollutants (cadmium and cephalexin) from microplastics from city tap water, seawater (to evaluate the impact on ecosystems), and stomachs of warm-blooded animals were simulated and studied. The results of this section are presented in Fig. 5. According to this figure, the percentage of desorption of both pollutants from microplastics in the stomach environment is higher than others, and seawater ranks next. The release of pollutants from microplastics depends on temperature, environmental pH, and surrounding ions. Recent reports have highlighted the harmful effects of microplastics on certain fish species, with evidence showing that polyethylene microplastics inhibited the catalytic activity of acetylcholinesterase within 96 h [71]. This occurred despite the absence of heavy metals and antibiotics in the microplastics. Therefore, humans and warm-blooded animals are at greater risk when they ingest microplastics, and the accumulation and risk of other pollutants are also included. In one study, the percentage of desorption of atrazine pollutants from polyethylene microplastic was reported to be more than 30 %, which is almost similar to the results of this study [42].

### 3.7 Human health risk assessment

Trace elements are naturally occurring minerals found in water sources. Excessive amounts of some trace elements can pose health risks to consumers who consume them through drinking water [72]. Heavy metals in water can enter the human body through ingestion, dermal contact, and inhalation [73]. In this study, the ingestion pathway was only considered. Our samples were real groundwater and distilled water (spiked with the desired amount of Cd, CEX, and MPs). In both samples, microplastics and cephalexin were added to the sample. To assess carcinogenic and non-carcinogenic effects associated with drinking water in children and adults, the daily intake dose of contaminants through drinking water was calculated (ADD_i_). For this purpose, the following formulas were used [73]. The hazard quotient (HQ) was also determined via the following equation:

(6) $$\text{HQ}=\frac{\text{ADDing}}{\text{RFDi}}$$

(7) $$\text{ADDi}=\frac{C\times \text{IR}\times \text{EF}\times \text{ED}}{\text{BW}\times \text{AT}}$$

(8) ELCR=ADDi×SFi

In ADD_i_, C denotes the concentration of Cd ions (mg/L), IR implies ingestion rate (L/day), EF is the exposure frequency (day/year), ED denotes exposure duration (year), BW implies body weight (kg), and AT is the average time (day).

The Excess Lifetime Cancer Risk (ELCR) associated with chemical contaminants is usually calculated using Eq. (8) [74]. SF indicates the carcinogenic slope factor, which is 0.38 mg/kg.day for cadmium [75]. RFD is the maximum amount of metal that has no chronic effect through drinking water and its amount according to EPA for cadmium is 0.0005 mg kg/day. The Delphi method is used to determine ELCR [76].

The carcinogenic and noncarcinogenic risks were computed for the Cd ions. In the case of non-carcinogenic risk, HQ in adults was greater than 1, and the probability of non-carcinogenic effects can occur in exposed populations. There is a medium risk of carcinogenesis from drinking groundwater (Table 6).

**Table 6:** The results of health risk assessment due to drinking water containing Cd, CEX, and MP.

Type of water	Conditions	Carcinogenic risk		HQ	
		Risk grades	Range of risk value	Child	Adult
Groundwater	Cd: 0.0053 mg/L, pH 6.7	5.7 × 10^−5^ Grade IV	Medium risk	0.70	3.02
	Cd: 0.004 mg/L, 1 g/L MP (30–63 μm), pH 6.7	4.7 × 10^−5^ Grade III	Low-medium risk	0.58	2.51
	Cd: 0.0038 mg/L, CEX: 1 mg/L, 1 g MP (30–63 μm), pH 6.7	4.1 × 10^−5^ Grade III	Low-medium risk	0.50	2.10
Distilled water	Cd: 0.0027 mg/L, pH 7.5	2.9 × 10^−5^ Grade III	Low-medium risk	3.6 × 10^−1^	1.54
	Cd: 0.0025 mg/L, 1 g/L MP (30–63 μm), pH 7.5	2.7 × 10^−5^ Grade III	Low-medium risk	3.3 × 10^−1^	1.42
	Cd: 0.0022 mg/L, CEX: 1 mg/L, 1 g/L MP (30–63 μm), pH 7.5	2.3 × 10^−5^ Grade III	Low-medium risk	2.9 × 10^−1^	1.10

In groundwater consumed in the rural areas of Iran, HQ values caused by water consumption in some rural areas were >1. The carcinogenic risk of cadmium for adults, children, and infants was greater than the safe level of 1.0 × 10^−4^ in the studied areas [77]. The carcinogenic risk is due to the long-term effects of exposure to pollutants and is presented in Table 6. In the groundwater samples, the amount of cadmium was greater than the international standard, which can be attributed to the possibility of contaminated wastewater entering the water. After adding MPs and CEX to water, the risk still exists. There is a low-medium risk and the risk of microplastics should also be considered.

## 4 Conclusions

The primary objective of this research was to assess the adsorption capacity of polyethylene (PE) microplastics for cadmium (Cd) in both single and combined systems with cephalexin (CEX). Cd uptake by PE microplastics increased as the pH shifted from acidic to neutral and decreased with rising salinity. The experimental data for adsorption were well-described by both PFO and PSO kinetic models, as well as Langmuir and Freundlich isotherms. The findings indicate that CEX negatively impacts Cd uptake by microplastics. Binding of Cd and CEX to PE microplastics did not alter the FTIR spectra of the microplastics. While the health risk of cadmium in aqueous solutions and groundwater was found to be low, it is important to consider the additional risk posed by microplastics. Desorption studies showed that the stomachs of warm-blooded animals can effectively remove larger quantities of pollutants from microplastics. These results are valuable for evaluating risks associated with the combined presence of microplastics and additives like Cd and CEX, and may aid in the development of strategies to mitigate pollution related to complex contaminants and microplastics in real-world environments. Future research could focus on the combined effects of microplastics and other additives on aquatic organisms and environmental health.

## CRediT authorship contribution statement

**Madineh Khoshmanesh:** Writing – original draft, Methodology, Investigation. **Ali Mohammad Sanati:** Supervision, Project administration, Investigation, Funding acquisition. **Bahman Ramavandi:** Writing – review & editing, Validation, Supervision, Methodology.

## Declaration of competing interest

The authors declare that they have no known conflict of interest that may obscure or affect their judgment to influence the work reported in this paper.
